# Reassessing the Prognostic Value of Point-of-Care Echocardiography in COVID-19 Patients: Right Heart, Wrong Signal?

**DOI:** 10.7759/cureus.99061

**Published:** 2025-12-12

**Authors:** David R Janese, Marshall Byun-Andersen, Jacquelyn Bowers, Phillip C Kilgore, Urska Cvek, Mary Ann Edens

**Affiliations:** 1 Emergency Medicine/Family Medicine, Louisiana State University (LSU) Health Shreveport, Shreveport, USA; 2 Emergency Medicine, Louisiana State University (LSU) Health Shreveport, Shreveport, USA; 3 Computer Science, Louisiana State University (LSU) Shreveport, Shreveport, USA

**Keywords:** bedside echocardiography, covid-19, d sign, emergency medicine, pocus, point-of-care ultrasound, right ventricular strain, sars-cov-2, transthoracic echocardiography, tte

## Abstract

Background

Right ventricular (RV) strain detected via transthoracic echocardiography (TTE) has emerged as a potential prognostic marker in patients with COVID-19, given the virus’s cardiovascular implications. However, data on the prognostic utility of point-of-care ultrasound (POCUS) in this context remains limited.* *This study evaluated whether RV strain identified through limited POCUS TTE at emergency department (ED) presentation correlates with adverse clinical outcomes or mortality in SARS-CoV-2 positive patients without pre-existing cardiovascular disease.

Methods

This study followed 29 patients at a medical center in the United States, in a prospective cohort design. Participants included patients who tested positive for COVID-19 via polymerase chain reaction (PCR) and had no history of myocardial infarction, congestive heart failure, percutaneous coronary intervention, pulmonary embolism, or atrial fibrillation. Each underwent bedside POCUS TTE to assess for RV strain. Follow-up was conducted via structured phone surveys at 30, 60, and 90 days using a four-question instrument developed by the study team. The study was conducted from initial enrollment through the final 90-day follow-up period, with data collected between September 2020 and August 2021. We used chi-square tests to examine the relationship between echocardiographic findings and clinical outcomes.

Results

Among those with RV strain (D Sign positive), 60% experienced adverse outcomes, compared to 73.7% in those without RV strain. This difference was not statistically significant (p = 0.7). Mortality was also lower in the RV strain group (10%) compared to those without RV strain (31.6%), though this difference did not reach statistical significance (p = 0.2).

Conclusion

In this preliminary cohort, RV strain identified via limited POCUS TTE was not significantly associated with adverse outcomes or mortality in COVID-19 patients without prior cardiovascular disease. To better understand whether bedside echocardiography can predict clinical outcomes for this group, more extensive research is necessary.

## Introduction

Since the emergence of SARS-CoV-2, clinicians have prioritized identifying efficient, reliable tools to stratify patients at risk for clinical deterioration. Although respiratory compromise has remained the primary focus, a growing body of evidence highlights the critical role of cardiovascular involvement in the morbidity and mortality associated with COVID-19. Notably, right ventricular (RV) strain may serve as an early sign of hemodynamic instability, particularly in individuals with no history of cardiovascular disease [1,2].

Point-of-care ultrasound (POCUS), especially when applied in the emergency department (ED), has gained traction for its ability to rapidly assess cardiac function. Transthoracic echocardiography (TTE) offers a practical, non-invasive approach for evaluating signs of RV strain, including dilation, hypokinesis, and septal flattening. While several observational studies and case reports have suggested associations between RV strain and adverse outcomes in COVID-19 patients, many have been limited by retrospective design, lack of control groups, or insufficient longitudinal follow-up [3-5].

Despite these preliminary findings, robust empirical data on the prognostic value of POCUS TTE in COVID-19 patients without existing cardiac disease remain scarce. This study sought to fill this gap through a prospective evaluation of whether RV strain detected via limited POCUS TTE at the time of ED presentation correlates with increased risk of adverse outcomes in SARS-CoV-2 positive individuals without pre-existing cardiovascular disease. The hypothesis was that, in individuals without existing heart conditions, a newly detected RV strain would lead to increased symptom progression or a higher risk of death over a 90-day follow-up period. Utilizing a longitudinal cohort design, the study integrated standardized follow-up protocols with structured interpretation of echocardiographic findings. Considering the potential of POCUS as a cost-efficient and real-time prognostic instrument, especially in periods of increased clinical demand, this study offers valuable insights into its application in frontline triage.

## Materials and methods

Study design and setting

This was a prospective, observational cohort study conducted at the ED of the Ochsner Louisiana State University Health Shreveport (O-LSUHS), a large academic medical center in Northern Louisiana, United States, between September 2020 and August 2021.. The goal was to assess whether RV strain detected by POCUS TTE could predict outcomes in patients positive for SARS-CoV-2 who had no previous cardiovascular disease.

Participants

Eligible patients were aged 18-100 years, tested positive for COVID-19 by polymerase chain reaction (PCR), and presented to the ED with symptoms consistent with viral syndrome (e.g., fever, malaise, anosmia, ageusia). Exclusion criteria included a history of myocardial infarction (MI), congestive heart failure (CHF), percutaneous coronary intervention (PCI), pulmonary embolism (PE), or atrial fibrillation (AF), as well as pregnancy or incarceration.

Procedure

Upon enrollment, each participant received a POCUS TTE. The ultrasound exam focused on four views: parasternal long axis, parasternal short axis, apical four-chamber, and subxiphoid. Findings suggestive of RV strain included RV dilation greater than two-thirds the size of the left ventricle, hypokinesis of the RV free wall with preserved apical function, and diastolic septal flattening. The presence of septal flattening-producing a characteristic "D-shaped" left ventricle in parasternal short axis view-was categorized as a “D Sign positive” finding, indicating positive right ventricular strain on echocardiography.

Each enrolled participant was followed for a period of 90 days following their initial ED visit. After undergoing a bedside echocardiogram during their index visit, participants were contacted at approximately 30, 60, and 90 days for outcome assessment. Research personnel conducting follow-up phone surveys were not blinded to POCUS finding. Data collection concluded following the final outcome call for all participants using the study-specific questionnaire (See Appendix A). The follow-up study-specific questionnaire was developed by the research team through expert consensus but was not validated against existing COVID-19 outcome instruments.

The composite outcome (‘poor outcome’) included both subjective measures-such as patient-reported worsening symptoms, persistence of initial symptoms, or development of new symptoms-and objective clinical events including return ED visit, hospitalization, or death. These components were combined into a single outcome category for analysis, although the mixture of subjective and objective elements limits the interpretability of the composite endpoint.

Mortality was confirmed through review of the electronic medical record (EMR) when available and supplemented by follow-up phone contact. Mortality was not determined solely based on patient or family report.

Sample size and data handling

**Figure 1:**
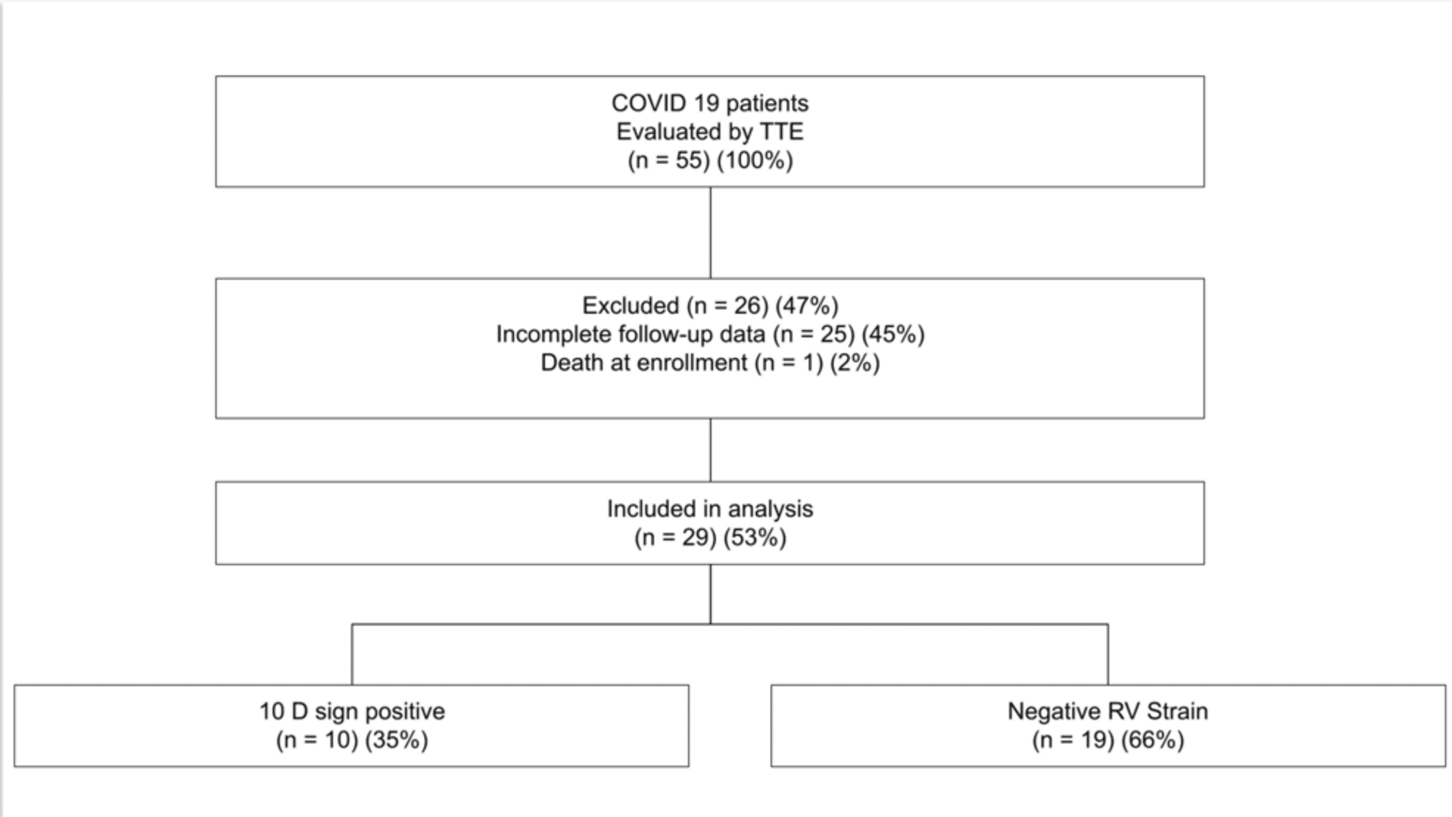
Cohort flow chart TTE: transthoracic echocardiography; RV: right ventricle

A total of 55 patients were initially enrolled. After excluding those with incomplete follow-up surveys, 29 (53%) patients remained for final analysis (Figure 1). The data underwent cleaning, standardization, and were then brought into R (R Foundation for Statistical Computing, Vienna, Austria, https://www.R-project.org/) for analytical purposes. All patient identifiers were deleted, and the data were kept in a database at LSUHS that complies with Health Insurance Portability and Accountability Act (HIPAA) regulations.

Participants were included in the final analysis if they completed at least two of the three follow-up surveys or if mortality data were available. Individuals with fewer than two follow-up contacts and no mortality confirmation were excluded from analysis. Missing responses within partially completed surveys were treated as missing at random and were not imputed

Statistical analysis

The chi-squared test was used to evaluate the association between TTE findings (D Sign positive vs. negative RV strain) and both clinical outcome and mortality. The outcome, whether it was poor or not, was represented as a binary variable. Statistical significance was defined as a p-value less than 0.05. However, this study was underpowered, with 29 (53%) subjects analyzed against a projected requirement of 88 for 80% power at α = 0.05 and a medium effect size [6].

## Results

Participant characteristics

The final analytic cohort consisted of 29 patients who completed at least two follow-up surveys or were deceased. Among these, 10 (34.48%) patients had echocardiographic findings indicative of right ventricular strain (D Sign positive), while 19 (66.52%) demonstrated negative RV strain findings. The majority were female (n=19, 65.51%) and 10 (34.48%) were male. Age was not collected as part of the study dataset. No other baseline demographic differences were available for comparison.

Prognosis and TTE findings

The primary outcome was defined as a poor prognosis based on self-reported symptoms (worsening condition, unresolved or new symptoms, or hospital readmission) or death. As shown in Table 1, 60% of patients with D Sign positive findings experienced a poor outcome, compared to 73.7% of those with negative RV strain. However, a chi-squared test indicated that this difference was not statistically significant (p = 0.7), suggesting that D Sign positive was not predictive of overall clinical prognosis.

**Table 1:** Prognosis vs. TTE findings (N=29) TTE: transthoracic echocardiography; RV: right ventricle

Outcome	D Sign + (n=10), n (%)	Negative RV Strain (n=19), n (%)
Good	4 (40.0%)	5 (26.32%)
Poor	6 (60.0%)	14 (73.68%)

Mortality

Of the 29 patients analyzed, seven (24.14%) were deceased by day 90. Mortality was lower among those with D Sign positive (1/10, 10%) than among those with negative RV strain (6/19, 31.58%). As illustrated in Table 2, this difference also did not achieve statistical significance (p = 0.2).

**Table 2:** Mortality vs. TTE findings (N=29) TTE: transthoracic echocardiography; RV: right ventricle

Mortality	D Sign + (n=10), n (%)	Negative RV Strain (n=19), n (%)
Alive	9 (90.0%)	13 (68.42%)
Deceased	1 (10.0%)	6 (31.58%)

Longitudinal symptom progression

To evaluate symptom progression over time, outcomes at 30, 60, and 90 days were compared. Among patients with D Sign positive, none experienced worsening between day 30 and day 60, while one patient with negative RV strain showed symptom worsening during that interval (Table 3). Four patients had missing values for either day 30 or 60 but completed other intervals.

**Table 3:** Day 30 vs. Day 60 0utcomes *One patient in the D Sign + group had incomplete day 30/60 data RV: right ventricle

Change in Symptoms	D Sign + (n=9)*, n (%)	Negative RV Strain (n=19), n (%)
Improved	9 (100.0%)	18 (94.74%)
Worsened	0 (0.0%)	1 (5.26%)

Summary of findings

In this prospective cohort study of 29 patients with confirmed COVID-19 and no prior cardiovascular disease, we found no statistically significant association between RV strain, as indicated by a D Sign positive on POCUS TTE, and adverse clinical outcomes. While 60% of patients with D Sign positive findings experienced poor outcomes, defined as symptom worsening, lack of resolution, emergence of new symptoms, or re-hospitalization, this was slightly lower than the 73.68% observed in patients with negative RV strain. Similarly, mortality was lower in the D Sign positive group (10.0%) compared to the negative RV strain group (31.58%). However, both differences failed to reach statistical significance, with p-values of 0.7 and 0.2, respectively.

Furthermore, longitudinal assessment of symptom progression from day 30 to day 60 showed that all patients in the D Sign positive group with complete data reported improvement (100.0%), while one patient (5.26%) in the negative RV strain group reported worsening symptoms. Again, this difference was minimal and not statistically significant. Overall, the data suggest that while RV strain may occur in COVID-19 patients, its presence on a single POCUS TTE evaluation does not reliably predict prognosis or mortality in patients without underlying cardiac conditions.

## Discussion

This prospective cohort study aimed to assess whether RV strain, identified via POCUS TTE, could predict poor outcomes in COVID-19 positive patients without pre-existing cardiovascular disease. Contrary to early hypotheses and several case-based reports, our findings suggest no statistically significant association between RV strain and poor prognosis or mortality.

In our cohort, 60.0% of patients with D Sign positive findings experienced poor outcomes, and 10.0% died during the 90-day follow-up period. Interestingly, 73.68% of patients with negative RV strain had poor outcomes, and 31.58% died. These differences were not statistically significant, with p-values of 0.7 and 0.2, respectively. Although these findings initially appear counterintuitive, they are not without precedent. RV dysfunction in COVID-19 has been described as multifactorial and sometimes transient, potentially leading to poor specificity as a prognostic indicator when used in isolation [6,7].

RV dysfunction has been associated with adverse outcomes in several retrospective and observational studies. For instance, Li et al. (2020) found that RV dilation and dysfunction were independently associated with increased mortality in hospitalized COVID-19 patients [8]. Similarly, Szekely et al. (2020) showed RV strain to be the most commonly observed cardiac abnormality in their cohort [9]. However, many of these studies included patients with known cardiovascular comorbidities, intensive care unit admissions, or required mechanical ventilation, making direct comparison with our lower-acuity, exclusion-based cohort difficult.

Our study aligns more closely with reports from Inciardi et al. (2020) [4] and Fried et al. (2020) [10], which documented cardiac abnormalities in COVID-19 but emphasized the heterogeneity of cardiac involvement. The absence of prognostic value in our findings suggests that POCUS TTE, while highly useful for diagnosis, may not independently predict disease trajectory in patients without cardiovascular risk factors.

Strengths and limitations

A major strength of this study is its prospective design, with standardized imaging criteria and structured symptom-based follow-up. This design allowed for consistent classification of outcomes using a pre-specified, investigator-developed questionnaire. The use of a single bedside imaging modality also reflects real-world emergency department practices, particularly during the resource-limited early stages of the pandemic.

However, the study had several limitations. Most importantly, the small sample size (n = 29) substantially limits statistical power, restricts generalizability, and increases the likelihood of Type II error. The outcomes questionnaire used in this study was investigator-developed and not previously validated, introducing potential measurement bias and limiting comparability to other COVID-19 outcome studies. Outcome assessment relied heavily on patient-reported symptoms and subjective perception of improvement or worsening, which may vary based on recall, health literacy, or response tendencies. The composite outcome combined subjective symptom measures with objective clinical endpoints such as hospitalization and mortality. This heterogeneity limits interpretability and may dilute associations between RV strain and clinically meaningful outcomes. Missing follow-up data also introduces the possibility of systematic bias, as 26 of 55 enrolled participants (47%) did not meet inclusion criteria for final analysis. If patients lost to follow-up differed meaningfully in symptom severity or disease progression, the estimates of adverse outcomes may be distorted. Another limitation is that RV strain was assessed at a single time point during the initial ED visit. Without serial imaging, transient or evolving RV dysfunction could not be tracked, which may obscure the relationship between dynamic cardiac changes and clinical trajectory [2,11].

Although previous studies have demonstrated RV dysfunction to be a marker of increased disease severity, our results suggest that isolated echocardiographic findings-particularly the D Sign-in patients without prior cardiac history may have limited utility as standalone prognostic tools. In emergency settings, clinical decisions should be informed by a combination of imaging, biomarkers (e.g., troponin, NT-proBNP (N-terminal pro-B-type natriuretic peptide)), and overall patient presentation.

Future research should focus on larger, multicenter trials that integrate point of care imaging with serial biomarker tracking, patient comorbidities, and treatment data. Incorporating artificial intelligence-driven echocardiographic interpretation may also help standardize assessments across sites and providers. Additionally, longitudinal studies with extended follow-up beyond 90 days may offer insights into the persistent cardiac sequelae seen in long-COVID syndromes [12].

## Conclusions

This prospective cohort study examined whether RV strain identified via POCUS TTE could predict poor outcomes in COVID-19 positive patients without pre-existing cardiovascular disease. Despite initial hypotheses and prior case reports suggesting a link between RV strain and clinical deterioration, our findings showed no statistically significant association between echocardiographic RV strain and either poor prognosis or mortality. Notably, while patients with negative RV strain experienced higher mortality rates in this cohort, the results were limited by small sample size and potential confounding factors. These findings suggest that isolated POCUS TTE results should not be used as standalone prognostic tools in this patient population. Instead, they should be interpreted in conjunction with comprehensive clinical evaluation and other diagnostic data. Given the non-invasive and accessible nature of bedside ultrasound, further investigation in larger, multicenter studies is warranted. Such research should incorporate serial imaging, biomarker correlation, and more robust statistical power to determine whether RV strain has a role in COVID-19 risk stratification and longitudinal care planning.

## Appendices

Patient questionnaire for 30/60/90 day (± 5 days) outcome

Study Number: Date of Initial Encounter: Date of Current Call Initial Encounter Outcome: Admission/Discharge 30-day outcome (If applicable): Admission/Supportive care/ED Visit for exacerbation 60-day outcome (If applicable): Admission/Supportive care/ED Visit for exacerbation 90-day outcome (If applicable): Admission/Supportive care/ED Visit for exacerbation

1. How do you feel as compared to when you were first diagnosed with COVID? Same/Better/Worse

2. Have the symptoms that you had when you were diagnosed with COVID 19 resolved? YES/NO

3. Are you having any NEW symptoms that you did not have when you were first diagnosed with COVID-19? YES/NO (If YES: List New symptoms as related to COVID-19)

4. Have you been to the Emergency Department or been hospitalized for worsening symptoms after your initial visit when you were diagnosed with COVID-19? YES /NO

Completed by name / date: ___________________________
